# Mental health of African asylum-seekers and refugees in Hong Kong: using the social determinants of health framework

**DOI:** 10.1186/s12889-016-3953-5

**Published:** 2017-02-02

**Authors:** William Chi Wai Wong, Sealing Cheung, Heidi Yin Hai Miu, Julie Chen, Kelley Ann Loper, Eleanor Holroyd

**Affiliations:** 1 Department of Family Medicine and Primary Care, The University of Hong Kong, 3/F., Ap Lei Chau Clinic, 161 Main Street, Ap Lei Chau, Hong Kong; 2 The Anthropology Department, The Chinese University of Hong Kong, Sha Tin, Hong Kong; 3 Bau Institute of Medical and Health Sciences Education, The University of Hong Kong, Pokfulam, Hong Kong; 4 The Department of Law, The University of Hong Kong, Pokfulam, Hong Kong; 5 Auckland University of Technology, Auckland, New Zealand

**Keywords:** Depression, Mental health, Social Determinants of Health, African refugees, Hong Kong

## Abstract

**Background:**

Hong Kong is non-signatory to the 1951 Refugee Convention and its 1967 Protocol, and has no systematic domestic policies committed to the rights of asylum-seekers and refugees (ASRs). This creates a tenuous setting for African ASRs there. This study explored how mapped social determinates of health has impacted the mental health and wellbeing of African ASR’s in Hong Kong.

**Methods:**

A cross-sectional survey was carried out with 374 African ASRs. The survey comprised of: (a) socio-demographics; (b) health status; (c) health behaviours; and, (d) social experiences. Associations between social determinants of health and depression screen were explored and multivariable regression analysis was conducted.

**Results:**

Majority of participants were 18–37 years old (79.7%), male (77.2%), single (66.4%) and educated (60.9% high school and above). Over a third (36.1%) screened positive for depression. Analyses revealed that living with family reduced the odds of a positive depression screen (OR = 0.25, 95%CI = 0.07–0.88). Those perceiving their health to be “Poor” were 5.78 times as likely to be screened for depression. Additionally, those with higher scores on the discrimination scale were more likely to have positive depression screen (OR = 1.17, 95%CI = 1.10–1.24).

**Conclusion:**

A significant proportion of African ASRs in Hong Kong exhibits depressive symptoms. A complex interaction combining both social and perceptions of health and discrimination in the host society is likely exacerbated by their ASR status. The use of community support groups or even re-examination of the family reunification laws could improve the mental health and wellbeing of African ASRs in Hong Kong.

## Background

### Refugees, asylum-seekers and torture claimants in Hong Kong

Every year thousands of people all over the world are displaced because of war, violence or persecution. The United Nations High Commissioner for Refugees (UNHCR) estimated that there were 51.2 million people who were forcibly displaced in 2014 [1]. Hong Kong, as a “hot spot” for transit, has over 10,000 asylum-seekers, refugees and torture claimants (ASRs) in 2016; approximately 10% of these are from the African continent [2, 3].

Historically, Hong Kong has resisted signing the 1951 Refugee Convention and 1967 Protocol [4, 5]; hence avoiding international legal instruments that establish rights and protections for refugees; including the right of non-refoulement, freedom of religious expression, rights to work, obtain housing, and education [6]. It was only officially recognised in 2013 that it is the duty of the Director of Immigration (DoI) to screen refugee and non-refoulment claims [7] leading to the formation of the Unified Screening Mechanism (USM). Previously, non-refoulment was granted by DoI, whilst refugee status was granted under a separate application to the UNHCR [8]. However, since its establishment, the recognition rate has been close to zero, leaving ASRs trapped in an indefinite limbo in Hong Kong [9]. Despite this, ASRs in Hong Kong are prohibited from working or studying (except those under the 18 years at the discretion of the Immigration Department) [5]. In 2013, at the time of the survey, ASRs received HK $1,200 (US $123) each month in rental assistance paid directly to their landlords, and food bags every 5–10 days, each containing HK $200–400 (US $16–32) worth of food until the next distribution [10]. These provisions were revised in 2014 to HK $1,500 rental allowance and HK $1,200 supermarket food coupons. This is still barely sufficient in a city known for its high cost of living. ASRs are often forced to live in desolate and inadequate conditions, sometimes without even access to drinking water [11]. Additionally, ASRs are required to obtain a waiver from the Social Welfare Department (SWD) before being allowed to access public healthcare services in Hong Kong.

This study focuses on African ASRs because of the limited knowledge base of African experiences and conditions, everyday life struggles, and their health concerns in Hong Kong. Given the society’s very minimal interaction with people from the African continent, African ASRs have been the source of intense scrutiny and racial discrimination. This subjects African ASRs to a distinct set of issues and concerns regarding their lives in Hong Kong, impinging directly on their physical and mental health.

### Refugees and mental health

The interweaving of mental health problems with the difficult life circumstances of ASRs is well-documented: displacement into unfamiliar environments can lead to depression, despair and sadness, disruption to the person’s ability to cope, regulate their mood, and interact with others [12, 13]. Poor mental health of ASRs can be the result of direct post-traumatic reactions to war and conflict, but also from post-displacement factors such as coming to terms with loss of family and home, cultural bereavement, and resettlement [12–15]. Stresses related to the latter commonly revolve around social alienation, discrimination, and racism [12, 15]. These are exacerbated by the confusion and frustration of adapting to a new socio-cultural environment [13]. However, the overarching socio-economic and political landscape of the host country can also have substantial impact on the mental health of ASRs. The negative effects of poor post-displacement accommodation, diminished status, and reduced employment opportunities are well recognised [14]. Many ASRs take on low-entry jobs, if any work at all, in order to support themselves [13], thus putting pressure on their physical and environmental resources, as well as exacerbating mental and emotional strain.

Thus, this study aimed to identify how different social determinants of health, within the political and socio-economic context of Hong Kong could impact on the mental health and well-being of the African ASR population.

## Methods

### Social determinants of health framework

**Fig. 1:**
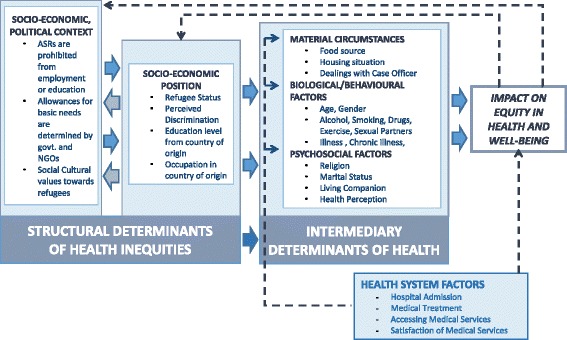
Mapping out of the Social Determinants of Health in the African ASR population in Hong Kong. Adapted from the WHO’s Conceptual Framework for the Social Determinants of Health (CSDH) framework (Solar & Irwin [16])

In 2010, the World Health Organization (WHO) Commission on Social Determinants of Health (CSDH) published a conceptual framework systematically mapping out determinants of health to structural intermediary and health system levels [16]. This framework was used to explore how mental health can be affected in the context of Hong Kong. For example, in Hong Kong, prohibition of ASRs from work or study created inequities manifesting in downstream/intermediary determinants of health, specifically; the reliance on food bags/food vouchers (Fig. 1). Although non-detrimental in the short run, the lack of transparency and low recognition rate of the USM traps and prolongs ASRs’ stay in Hong Kong, hence amplifying and exacerbating these problems. Further, the need for ASRs to obtain a Medical Waiver from the SWD contribute additional barriers to access of healthcare in addition to the language and cultural barriers that are already problematic in Hong Kong’s healthcare system. Both of these above examples are only further aggravated by prevalence of discrimination towards ASRs from the local populations (Fig. 1).

### Study design, setting and participants

The present analysis was part of a larger research project on the African ASR population in Hong Kong. It encompassed a cross-sectional survey carried out between February and April 2013 which was developed through an initial explorative study using literature reviews, observations, and in-depth interviews with African ASRs living in Hong Kong. This was done to elicit contextually relevant personal narratives of the targeted population; including health and social issues. Only analysis of the cross sectional study will be detailed in this paper. Recruitment of participants, through convenience sampling was carried out in collaboration with three local organisations; The Vine Church, Vision First and the African Community Centre who regularly provide legal advice, education, and other services to ASRs. The collaboration meant that sampling an otherwise hidden population was much more feasible and information obtained more trustworthy. Researchers and outreach workers, briefed and trained for data collection, invited African ASRs aged 18 years or above, able to read and write in English, French, or Somali. Our inital explorative study with members of the African communities indicated that aside from some Somali speaking members, all in the community should be fluent in either English or French. Participants were informed of the purpose of the study, provided clear explanations of their confidentiality policy, and their rights to withdraw. They were also pre-screened for psychological fitness to participate and an advisory committee consisting of non-governmental organisation (NGO) representatives, legal and health experts, and researchers was set up to safeguard interest of respondents. Participants were requested to sign a written consent prior to the study and a cash incentive of HK $50 (US $6.45) was given as remuneration.

Based on the paper that assessed HIV knowledge of African asylum-seekers lived in Europe, their knowledge was about 69%. Assuming the power of 80% and standard deviation of 0.05, the estimated sample size will be 328. In total, 374 participants were recruited.

### Survey instrument

The survey comprised of 55 questions seeking information across four domains: a) socio-demographics; b) health status; c) health behaviours; d) and social experiences in Hong Kong (Table 1.). Originally drafted in English, the survey instrument was pilot-tested for face validity with eight African ASRs, who would not participate in the main study, revisions were made accordingly. This was then translated to French or Somali, proofread and edited by two native French speakers or back-translated by two coordinators of the African Community Centre respectively. Health perception was included as a means to assess their bio-social concepts to health, for example participants were asked whether they perceived their health to be better, same, or worse compared to Hong Kong local residents of the same age (“health vs locals of same age”).

**Table 1:** Everyday Discrimination Scale (Short) [17–19]

Consisting of 5 items, the scale assessed frequencies of different day-to-day mistreatment; with a Cronbach alpha of 0.77. Participants were asked to rate the frequency of the following items:
1. You are treated with less courtesy or respect than other people.
2. You receive poorer service than other people at restaurants or stores.
3. People act as if they think you are not smart.
4. People act as if they are afraid of you.
5. You are threatened or harassed.
Responses were rated into: never, less than once a year, a few times a year, a few times a month, at least once a week and multiple times a week

#### Everyday discrimination scale

A shortened version of the Everyday Discrimination Scale (EDS) (Table 2) to assess frequencies of different forms of mistreatment and discrimination [17] amongst African ASRs in Hong Kong, in accordance to their own subjective experiences [18, 19]. This scale consists of 5 items probing frequency of discrimination on a six-point scale (0 = never, 1 = less than once a year, 2 = a few times a year, 3 = a few times a month, 4 = at least once a week, 5 = multiple times a week). There scores were combined into a final score for analysis (Refer to Table 3.).

**Table 2:** Basic descriptive of African ASRs in Hong Kong, assessing different demographic factors and intermediary determinants of health as defined by the CSDH framework (Solar & Irwin [16]). (*n* = 374)

Socio-demographics characteristics	
Gender	
Male	292 (78.1%)
Female	82 (21.9%)
Age Group (Mean: 31.52, SD: ±7.41)	
18–27years	107 (28.6%)
28–37years	194 (51.9%)
38–47years	66 (17.6%)
48years +	7 (1.9%)
Place of origin	
North Africa	13 (3.5%)
East Africa	131 (35.0%)
Central Africa	69 (18.4%)
South Africa	10 (2.7%)
Western Africa	151 (40.4%)
Length of Residence in Hong Kong	
Less than 5 years	280 (74.9%)
5 to 10 years	89 (23.8%)
11years plus	5 (1.3%)
Intermediarydeterminants of health	
Material/Living Circumstances	
Problems with Accommodation	
No	80 (21.4%)
Yes	294 (78.6%)
Problems with food packages	
No	132 (35.3%)
Yes	242 (64.7%)
Problems with case officer	
No	174 (46.5%)
Yes	200 (53.5%)
Biological/Behavioural Circumstances	
Alcohol consumption^a,b^	
None	268 (71.7%)
Infrequently	57 (15.2%)
Frequently	49 (13.1%)
Smoking^a,c^	
None	260 (69.5%)
Light Smoker	24 (6.4%)
Heavy Smoker	90 (24.1%)
Recreational Drugs^a^	
No	342 (91.4%)
Yes	32 (8.6%)
Exercise^a,d^	
Little or none	276 (73.8%)
Frequent	98 (26.2%)
Multiple Sex partners^a^	
None	201 (53.7%)
Only one	117 (31.3%)
More than one	56 (15.0%)
Illness, Injury, or symptoms^a^	
No	193 (51.6%)
Yes	181 (48.4%)
Chronic illness	
No	223 (59.6%)
Yes	151 (40.4%)
Psychosocial Factors	
Religion	
Atheist	14 (3.7%)
Christian/Catholic	211 (56.4%)
Muslim	142 (38.0%)
Other	7 (1.9%)
Marital Status	
Single	252 (67.4%)
Married	71 (19.0%)
Divorced/Separated/Widowed	51 (13.6%)
Living Companion	
Alone	286 (76.5%)
With Family	65 (17.4%)
With Others	23 (6.1%)
PHQ-2 Score (Cut off at 2)	
Below 2	239 (63.9%)
Equal or above 2	135 (36.1%)
General health^e^	
Very Good/Excellent	72 (19.2%)
Good	114 (30.5%)
Fair/Poor	188 (50.3%)
Health vs. locals of same age^e^	
Better or Much Better	162 (43.3%)
Same	133 (35.6%)
Worse or Much Worse	79 (21.1%)
Health vs. prior arrival in HK^e^	
Better or Much Better	148 (39.6%)
Same	110 (29.4%)
Worse or Much Worse	116 (31.0%)
PHQ-2 Score (Cut off at 4)	
Below 4	327 (87.4%)
Equal or above 4	47 (12.6%)

^a^ Reported in the past 30 days; ^b^ Alcohol Consumption – None = does not drink, infrequently = <5 drinks/week, Frequently = >5 drinks/week; ^c^ Smoking – Light Smokers = <10 cigarettes per day, Heavy Smokers = >10 Cigarettes per day; ^d^ Exercise – Little or none = <15 days with exercise, Frequent = >15 days with exercise; ^e^ Based on the participants own judgement

**Table 3:** Basic descriptive of African ASRs in Hong Kong, assessing structural and health system determinants of health as defined by the CSDH framework (Solar & Irwin [16]) (*n* = 374)

Health system determinants of health	
Satisfaction with Medical Services	
Not used	87 (23.3%)
Fair/Poor	105 (28.1%)
Good-Excellent	182 (48.6%)
Admission to hospital^a^	
Yes	146 (39.0%)
No	228 (61.0%)
Difficulties Accessing Medical Facilities	
No	104 (27.8%)
Yes	270 (72.2%)
Obtained treatment due to illness/injury^a^	
Yes	217 (58.0%)
No	157 (42.0%)
Structural determinants of health	
Socio-economic Position	
ASR Status	
Refugee	32 (8.6%)
Asylum Seeker Claimant	172 (46.0%)
Torture Claimant	115 (30.7%)
Asylum Seeker & Torture Claimant	55 (14.7%)
Education Level in country of origin	
None	55 (14.7%)
Primary	87 (23.3%)
High School	177 (47.3%)
University or above	55 (14.7%)
Occupation in country of origin	
Professional	124 (33.1%)
Supervisory	57 (15.2%)
Skilled manual worker	74 (19.8%)
Semi/Unskilled manual worker	44 (11.8%)
Casual worker or unemployed	75 (20.1%)
Discrimination	
Everyday Discrimination Scale: Mean = 12.87; SD = ±7.01	
Treated with less respect than locals^a^	
None	118 (31.5)
Not regularly	96 (25.7)
Regularly	160 (42.8)
Receive Poorer Services than locals^a^	
None	104 (27.8)
Not regularly	83 (22.2)
Regularly	187 (50.0)
Considered Not Smart^a^	
None	100 (26.8)
Not regularly	88 (23.5)
Regularly	186 (49.7)
People Fear you^a^	
None	117 (31.3)
Not regularly	84 (22.4)
Regularly	173 (46.3)
Felt threatened^a^	
None	169 (45.2)
Not Regularly	79 (21.1)
Regularly	126 (33.7)

^a^Reported in the past 12 months

#### The patient health questionnaire-2

The Patient Health Questionnaire-2 (PHQ-2) was used to screen for depression. Validated for such purpose [9, 20], participants are asked whether they have had a) experienced little pleasure in doing things; and b) experienced feeling down, depressed or hopeless in the past two weeks. Responses are scored from 0 to 3 from: not at all, several days, half of the days, to nearly every day respectively. For clinical screening purposes, participants scoring 2 or more were considered to screen positive for depression [9].

### Data analysis

Data cleaning and analysis was done using SPSS (Version 20.0). Survey variables were mapped to CSDH [16], specifically according to the structural, intermediary and health system factors (Fig. 1.). Frequencies, crude odds ratios (OR) for univariate analysis, and adjusted odds ratios (aOR), for multivariable analysis, were assessed to explore different associations to PHQ-2 using a cut-off point of 4 (Positive screen = score of <4) for enhanced specificity. Processing of different types of variables were processed accordingly, e.g. continuous (e.g. age and discrimination scale) and categorical variables. For the latter, reference groups for categorical variables are noted in the tables next to the variables. All assumptions of linearity were made and, normally distributed and uncorrelated errors were checked and met.

## Results

### Socio-demographics and social experiences of African ASRs in Hong Kong

Participant demographics and background characteristics can be found in Tables 2. and 3. Majority of the 374 participants were male (*n* = 292; 78.1%) and single (*n* = 252; 67.4%). With a mean of 31.52 years (Standard Deviation (SD) = ±7.41), majority of participants were under 37 years (80.5%). Prior to their arrival in Hong Kong, 181 (48.3%) had professional and supervisory roles, with 232 (62%) also having obtained high school education or above. Using the PHQ-2 screening tool, a surprising 135 (36.1%) of participants scored equal to or above 2; indicating of a positive clinical depression screening. At a cut-off point of 4, 47 (12.6%) of participants screened positive. With regards to their health, 181 (48.4%) and 151 (40.4%) have suffered from illness or injury in the past 30 days or reported chronic illness respectively.

In Hong Kong, 286 participants lived alone (76.5%), whilst 88 lived either with family or others (23.5%). Up to 242 (64.7%) of participants reported problems with their food bags; 294 (78.6%) highlighted housing-related problems; and 200 (53.5%) cited problems with the case officer who managed their refugee application. Of those that had used the healthcare system, 182 (48.6%) reported the services to be “Good” to “Excellent”, however a large majority, 270 (72.2%), reported having difficulties accessing medical facilities, particularly with obtaining the medical waiver. The EDS gave a combined mean score of 12.87 (SD = ±7.01). Participants regularly experienced receiving poorer services than locals (*n* = 187; 50.0%); considered to “be not smart” (*n* = 186; 49.7%); or felt that others feared them (*n* = 173; 46.3%) (Table 3).

### Intermediary determinants of depression

Those who lived with their families had reduced odds of a positive depression screen compared to those living alone; with an adjusted OR (aOR) of 0.25 and a 95% confidence interval (95%CI) of 0.07–0.88 upon adjusting for other variables using multivariable regression (Table 4). Those who lived with others, such as friends, also saw reduced chance of screening positive for depression (aOR = 0.97, 95%CI = 0.23–1.04), although not statistically significant. In addition, self-reported health also revealed that those who perceived their health to be “poor” were 5.78 times as likely to be screened as positive for depression compared to those rating their health to be “very good/excellent”.

**Table 4:** Crude Odds Ratio (OR) and adjusted Odds Ratio (aOR) analysis of variables significant to positive depression screen using the PHQ-2 on African ASRs in Hong Kong

Variable	OR	95%CI	aOR	95%CI
Intermediary determinants of health				
Age	1.05	(1.01–1.09)*	1.02	(0.97–1.07)
Exercise^a,b^	0.37	(0.15–0.91)*	0.42	(0.15–1.15)
Illness, Injury, or symptoms^a^	2.55	(1.33–2.17)*	1.23	(0.49–3.06)
Chronic illness	2.44	(1.31–4.56)*	0.87	(0.35–2.18)
Living Companion (Alone)				
With Family	0.30	(0.09–0.99)*	0.25	(0.07–0.88)*
Others	1.29	(0.41–4.00)	0.97	(0.23–4.04)
General health ‖ (Very Good/Excellent)				
Good	2.46	(1.66–9.13)	3.18	(0.71–14.19)
Fair/Poor	4.90	(1.45–16.51)*	5.78	(1.40–23.81)*
Health vs. locals of same age^c^‖ (Better)				
Same	2.65	(1.30–5.42)*	2.22	(0.90–5.49)
Worse	1.49	(0.60–3.61)	0.63	(0.20–1.98)
Healthsystem determinants of health				
Reported difficulties accessing Med Services (Yes)	2.92	(1.20–7.11)*	3.57	(0.95–13.44)
Satisfaction with Medical Services (Not Used)				
Good - Excellent	1.27	(0.43–3.71)	0.75	(0.16–3.41)
Fair - Poor	2.88	(1.16–7.18)*	0.92	(0.23–3.67)
Obtained treatment due to illness/injury^d^	2.32	(1.16–4.64)*	1.19	(0.44–3.25)
Structural determinants of health				
Everyday Discrimination Scale^d^	1.17	(1.11–1.23)**	1.17	(1.10–1.24)**

^a^ Reported in the past 30 days; ^b^ Exercise – Little or none = <15 days with exercise, Frequent = >15 days with exercise; ^c^ Based on the participants own judgement; ^d^ Reported in the past 12 months. ** *p*<0.01; * *p*<0.05

### Health system and structural determinants of depression

ARS who reported difficulties accessing medical services showed an increased odds for depression when assessed independently (OR = 2.92, 95%CI = 1.20–7.11) compared to no reported difficulties when not adjusted (Table 4.). EDS also showed association whereby an increase in self-reported discrimination was accompanied by increase in odds of a positive depression screen (aOR = 1.17, 95%CI = 1.10–1.24) (Table 4.).

## Discussion

To our knowledge, this is the first study to assess the mental health of African refugees residing in Hong Kong. Unique to this study is the assessment of the prevalence of depression and the determinants associated with depression in this population. Using the PHQ-2 depression screening tool, the study identified the prevalence of clinical depressive symptoms to be as high 36.1%. This is notably higher when compared to the general population of Hong Kong; found to be 10.7% in patients of primary care facilities [21]; suggesting an increase burden of mental stress of African ASRs. Other studies exploring depression in African ASR populations elsewhere, whilst using different instruments, have also noted similar rates of depression ranging between 30 and 40% [22, 23].

The nature of internal displacements of persons has been demonstrated to increase likelihood to mental health problems with many studies addressing the potential of developing depression from post-migration resettlement [24, 25]. Using the CSDH [16] framework, this study shows that the presence of family members and friends can be associated with a reduced chance of depression compared to being alone. This is likely be associated with increased social support, defined as “*interactions between family members, friends, peers… and professionals that communicate information, esteem, practical, or emotional help…*“ [26]. The impact of family as well as other forms of social support has been deemed an important part of the post-migration resettlement process [27]. Commonly participants harbour heightened concerns for the wellbeing of family members in the host country; whereby uncertainty is often created from limited contact with family members, causing stress from prolonged and indefinite separation [24].

Considering that majority of the African ASR population in Hong Kong lived alone, this would suggest that the lack of much family support places them at a heightened risk for mental health illnesses. The financial restraints on ASRs in Hong Kong incur limited funds for housing and food, creating considerable stress on everyday life. In addition, there is also the limited government support for transportation or social activity [28]; not to mention allowance for buying phones or other means of communication. This only further restricts the amount social support available from friends, community members, as well as connections with those back home. In fact, the importance of social support and poor post-migration circumstances on mental health has also been demonstrated amongst of newly arrived migrants from Mainland China [29]. The effect of such social deprivation not only engenders a mounting sense of alienation for these informants who predominately live on their own, but such circumscription of their social networks could further undermine their access to available services and help more effectively [30].

Self-reported health has frequently been used as a measure of health, considered to integrate an individual’s biological but also psychological and social dimensions of health [31]. Analysis shows that those who rated their health as poor were more than five times more likely to be screened positive for depression. Vonnahme et al. [32] found that Bhutanese refugees reporting poor health were highly associated with depression. Further, the restricted lifestyles of ASRs in Hong Kong may prolong ill health when medical access is hindered or culturally insensitive.

Self-perceived discrimination, in addition to the intermediary factor, appeared to inform self-reported depression in African ASRs residing in Hong Kong. Discrimination is a significant post-migration barrier for refugees [33, 34], and along with racial identity has been linked to depressive symptoms [35]. Very limited research to date has assessed the discrimination of African ASRs in Hong Kong or China. However, Bodomo [36] contends that treatment of African travellers at the Chinese border is based on racial perceptions rather than due to language or cultural misunderstandings. This finding is further anecdotally supported by social media that perceptions of “local” and “belonging” can be a problem faced by ethnic minorities in Hong Kong [37]. Further aggravating this situation is the relatively hidden visibility of ASRs in Hong Kong. More research is needed to explore the racial perceptions Hong Kong and Chinese local resident have towards ASRs, particularly those of African origins.

### Implications

The high prevalence of depression found in the African ASR population contended that full mental health assessment should be made available upon arrival to Hong Kong and regularly reviewed. Research has highlighted the need for mental health services which are not only culturally appropriate but also aware of the post-migration circumstances and health determinants further propagating vulnerability to mental health problems [25]. Through exploring the social determinants of health, the need for increased social support was clearly evident. Establishing grassroots community and social support groups for ASRs has potential to bring positive effects through multiple channels. Firstly, as many African ASRs live alone, community groups with sufficient support from the government could be an alternative option to enhance their social network in providing a platform for psychological and emotional support. Secondly, this platform can also explore the possibility of setting up alternative health and mental health services as currently exist for certain marginalised groups such as female sex workers [38]. These services could be combined with health and social services [25], as well as providing self-empowerment programmes for ASR populations on health and social issues. Thirdly, to address the discrimination, the use of community and social groups can enhance opportunities for community engagement through community fairs or events in public spaces [30]. This can enable interactions with local populations, helping to break down negative public stereotypes of an otherwise invisible population. Finally, the grossly inadequate support provided by the Hong Kong government, together with the restrictive conditions of stay as ASRs, and the prolonged wait of their applications (sometimes more than a decade) have created conditions that have significantly negative impact on their mental health.

### Study limitations

Limitations of this study included the use of convenience sampling of African ASRs aged 18 years or above, who were able to read and write in English, French, or Somali. Although the choice of languages used were based on the initial explorative study, this may bring bias due to exclusion of African ASRs speaking other languages as well as ASRs who do not make use of NGO’s for services. Hence caution should be taken on generalisability of results. However no incidences of exclusion due to language limitations were reported. Secondly, the use of a cross-sectional survey design and the statistical analysis used meant that despite the ability to assert associations between factors and outcomes conclusions about the directionality and causation cannot be made; results must be considered and interpreted accordingly. To further understand the directionality of causation, there is a need for more in-depth and longitudinal studies. Finally, the use of PHQ-2 was designed as an initial screening mechanism. More comprehensive diagnostic tools could be used for more specific diagnosis of depression or other mental illnesses.

## Conclusion

The study has revealed that a significant number of African ASRs in Hong Kong exhibit depressive symptoms. This has been significantly associated with lack of family and social support; further exacerbated by their status as ASRs in Hong Kong which imposes a financially restrictive lifestyle and limited healthcare access. Related to this, the study suggests that poor perceived health also exacerbates mental stressors of African ASRs. In addition, reports of discrimination were also linked to an increase in depressive symptoms, suggesting a need to for more research into racial perceptions in Hong Kong residents and citizens. The use of community support groups combined with changes to the family reunification laws would enhance social support networks and access to resources to African ASR who are often caught indefinitely awaiting assessment of their refugee status in Hong Kong.
